# Association Between School-Related Google Trends Search Volume and Suicides Among Children and Adolescents in Japan During 2016-2020: Retrospective Observational Study With a Time-Series Analysis

**DOI:** 10.2196/51710

**Published:** 2024-10-21

**Authors:** Takahiro Arai, Hiroe Tsubaki, Ayako Wakano, Yasuyuki Shimizu

**Affiliations:** 1 School of Management and Information Sciences Tama University Tokyo Japan; 2 Graduate School of Health Management Keio University Kanagawa Japan; 3 Japan Suicide Countermeasures Promotion Center Tokyo Japan; 4 The Institute of Statistical Mathematics Tokyo Japan; 5 Department of Economics, School of Political Science and Economics Tokai University Kanagawa Japan

**Keywords:** adolescent, children, COVID-19, Google Trends, internet, Japan, monitoring, suicide, surveillance, time series analysis

## Abstract

**Background:**

Suicide is the leading cause of death among children and adolescents in Japan. Internet search volume may be useful in detecting suicide risk. However, few studies have shown an association between suicides attempted by children and adolescents and their internet search volume.

**Objective:**

This study aimed to examine the relationship between the number of suicides and the volume of school-related internet searches to identify the search terms that could serve as the leading indicators of suicide prevention among children and adolescents.

**Methods:**

We used data on weekly suicides attempted by elementary, middle, and high school students in Japan from 2016 to 2020, provided by the National Police Agency. Internet search volume was weekly data for 20 school-related terms obtained from Google Trends. Granger causality and cross-correlation analysis were performed to estimate the temporal back-and-forth and lag between suicide deaths and search volume for the related terms.

**Results:**

The search queries “I do not want to go to school” and “study” showed Granger causality with suicide incidences. The cross-correlation analysis showed significant positive correlations in the range of –2 to 2 for “I do not want to go to school” (highest value at time lag 0, *r*=0.28), and –1 to 2 for “study” (highest value at time lag –1, *r*=0.18), indicating that the search volume increased as the number of suicides increased. Furthermore, during the COVID-19 pandemic period (January-December 2020), the search trend for “I do not want to go to school,” unlike “study,” was highly associated with suicide frequency.

**Conclusions:**

Monitoring the volume of internet searches for “I do not want to go to school” could be useful for the early detection of suicide risk among children and adolescents and for optimizing web-based helpline displays.

## Introduction

In Japan, suicide is the leading cause of death among people aged 10-19 years [1]. Suicide among children and adolescents has continued to increase since 2016, with data for 2020 showing the suicide rate (per 100,000 population) for primary, secondary, and high school students totaled 4.0, with 0.2 for primary school students, 4.5 for secondary school students, and 11.0 for high school students. One of the key reasons for suicide is school-related problems [2]. Therefore, suicide prevention among children and adolescents is primarily conducted in schools. However, school-based suicide prevention programs have exhibited limited effectiveness in reducing suicide attempts and rates [3,4]. In recent years, suicide prevention using the internet has received greater attention to complement the effectiveness of school-based suicide prevention programs directed toward the young population. As the internet has become a prevailing medium for young people, suicide prevention has been examined using internet spaces rather than mental health care in schools and gatekeeping using the phone [5]. Recent studies have analyzed the association between the volume of suicide-related terms derived from internet search queries and suicide [6-11].

The field of public health has explored the development of monitoring methodologies using internet searches. During the COVID-19 pandemic, there have been suggestions that Google Trends could be used to aid disease surveillance systems [12-14]; their complementary function has also been explored in the context of suicide prevention [15,16].

The Japanese government focused on the relationship between internet search queries and suicide and proposed a suicide prevention strategy using internet search mechanisms in 2017 [17]. However, the empirical findings were quite limited. Sueki [18] reported a significant positive correlation between the number of suicides and the search term “utsu” (depression). Hagihara et al [19] further reported a strong association between suicide rates and the search term “hydrogen sulfide,” a means of attempting suicide. Both studies covered the period before the COVID-19 pandemic, and search terms were related to mental illness and crisis interventions, such as the means of attempting suicide. Furthermore, during the COVID-19 pandemic, Taira et al [20] found that keywords such as “no money” and “divorce” could predict the number of suicides, discovering a new linguistic marker by targeting search terms related to social factors rather than words directly related to specific disease names or suicide.

Internet search terms may provide clues for identifying suicide crisis pathways and be useful for optimizing web-based suicide prevention [21]. However, the research investigating an association between suicide deaths among children and adolescents and internet search queries is scant. Therefore, new linguistic markers that have the potential to detect suicide risk among children and adolescents need to be explored.

Our study aims to identify the search terms that could serve as the leading indicators of suicide among children and adolescents. For this purpose, we investigated the relationship between the number of suicides and internet search volumes. We further examined how these trends changed in the social context during the COVID-19 pandemic. In this study, children and adolescents are defined based on their school attendance at elementary, junior high, and high schools rather than by age. This definition, as used in the school education system, is considered a prominent factor contributing to the occurrence of suicide among children and adolescents in Japan [2].

Several analytical challenges regarding the usefulness of search queries in suicide prevention remain unresolved. In other words, the coarseness of the monthly data on the number of suicides is problematic. To tackle this weakness, we propose that using weekly data can change the analysis results [22]. Our study has 2 unique features. First, we used weekly data on the number of suicides and internet search volumes because monthly analysis may not observe changes in highly fluid social conditions. Second, we used the number of suicides attempted by elementary, middle, and high school students rather than the total number of suicides. The Japanese suicide surveillance system includes occupational status.

## Methods

### Suicide Data

The National Police Agency provided data on the number of suicides. These were weekly data processed by the Japan Suicide Countermeasures Promotion Center under the supervision of the Ministry of Health, Labor, and Welfare after receiving confirmation from the National Police Agency. The data collection period was 5 years, from 2016 to 2020, and the analysis included elementary, middle, and high school students. The variables present in the National Police Agency data are demographic variables, gender (binary), age (integer), occupational status (51 categories including elementary, middle, and high school students), living together (3 levels—yes, no, and unknown), municipality of residence (character), suicide municipality (character), and other or not having a history of suicide attempts (3 levels—yes, no, and unknown). Selecting participants solely based on age would not adequately exclude the individuals who dropped out or did not attend high school after graduating from their junior high school. Therefore, this study used occupational status categories to accurately identify and include elementary, junior high, and high school students.

### Internet Search Data

Search queries used weekly data (7-day interval from Sunday to Monday) from January 3, 2016, to December 26, 2020, using Google Trends. The Google Trends search volumes during this period were normalized between 0 and 100. According to Google Trends, the normalization method is described as “search results are normalized to the time and location of a query by the following process: Each data point is divided by the total searches for the geography and time range. The resulting numbers are then scaled on a scale of 0 to 100 based on a topic’s proportion to all searches on all topics” [23]. Therefore, it only shows a relative value rather than a pure search frequency. Relative search values (RSV) are calculated as follows.

For the search volume of a word “*i*” in a certain week “*t*,” in a certain region “*j*,” let *Qmax_i,j_* be the maximum search volume when it was the maximum in all periods.


*Qmax_i,j_=max{search_i,j,t_}*

**(1)**

The amount of retrieval of the search word “*i*” in a given week “*t*” is relative to the maximum value.



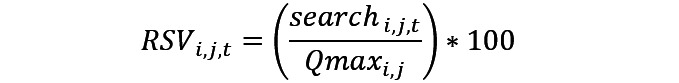



**(2)**

The search terms used in this study were selected based on a list of the causes of student suicide [2]. We excluded “suicide” and “I want to die,” which have been used in many studies, because they are likely to be searched by many people unrelated to suicide and are not necessarily representative of suicide risks among children and adolescents [24]. In addition, we integrated words related to school resumption because the school resumption timing is considered to cause strong psychological stress among young people [25].

Based on the above information, 20 keywords were selected for this study, such as “school,” “school reopening,” “I do not want to go to school,” “school hard,” “school anxiety,” ”When is school?” “When does school start?” “school start,” “school bullying,” “school friends,” “school grades,” “bullying,” “friendships,” “study,” “anxiety about studying,” “I do not know how to study,” “mid-term or term-end exam,” “entrance examination,” “future career,” and “grades.”

### Statistical Analysis

**Figure 1:**
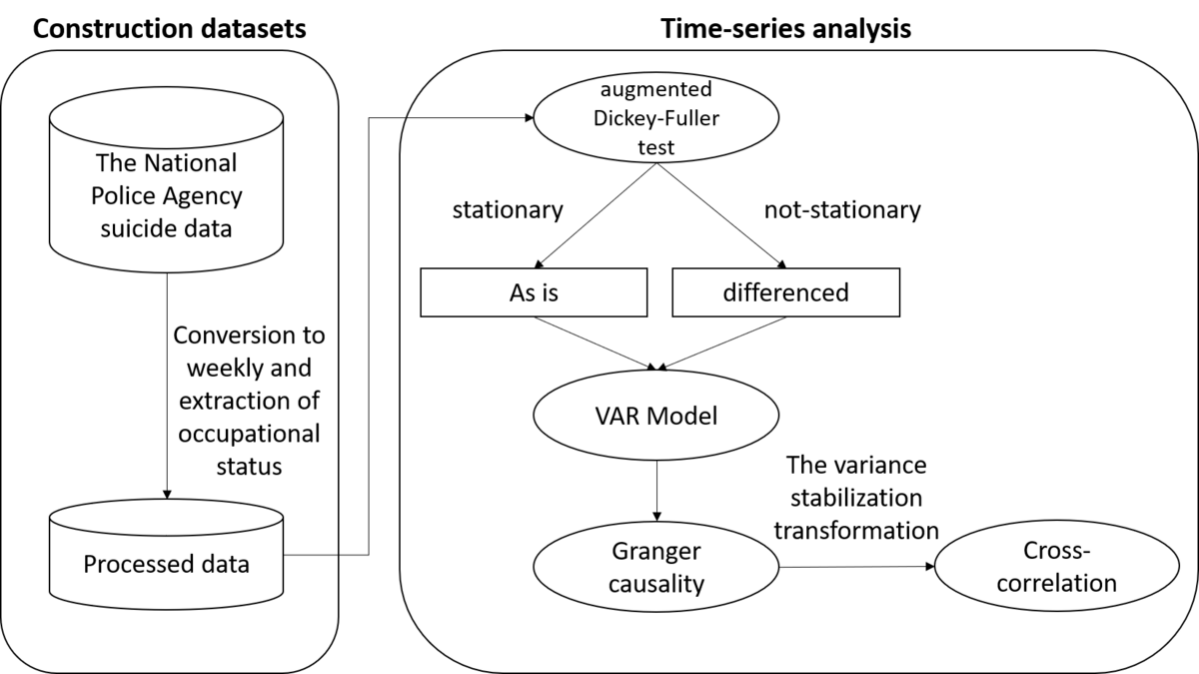
Concept map expressing the methodology of time series analysis. VAR: vector autoregressive.

We used the time-series analysis approach used in previous studies [26]. Referring to previous studies, we constructed a concept map to vividly illustrate time-series analysis (Figure 1).

First, a unit root test (an augmented Dickey-Fuller [ADF] test) was performed on the collected time-series data (n=260 weeks) using the R package (R Core Team) “tseries” [27] to examine whether the sequence was stationary or not. Stationarity is an important assumption in many statistical analyses, including Granger causality analysis. The null hypothesis of the ADF test (H0) assumes the existence of a unit root and indicates nonstationarity, while the alternative hypothesis (H1) indicates stationarity. With the significance level set at .05, a *P* value below it justifies the rejection of H0 and affirms the stationarity of the data. Next, the vector autoregressive (VAR) model was estimated using the R package “vars” [28]. It incorporates the optimal lag variables, whose order was determined by Akaike’s information criterion (AIC), and the Granger causality was verified. Granger causality analysis is a statistical method used to determine if one time-series variable can predict another time-series variable. To assess the significance of the relationship, we performed the *F* test. If the *P* value was below the significance level (*P*<.05), we rejected the null hypothesis and concluded that there is evidence of Granger causality. However, if the *P* value was above the significance level, we failed to reject the null hypothesis, indicating that there was no evidence of Granger causality. Furthermore, to adjust for the influence of the school calendar, we incorporated dummy variables for weeks including April 8, September 1, and January 8, which are anticipated to have spikes in suicide risk as they mark the end of spring, summer, and winter breaks, respectively [25]. We corrected the number of suicides using these dummy variables in our Granger causality testing model (the school calendar model). We tested the Granger causality test using this adjusted number of suicides.

The Google Trends model for the Granger causality test is as follows.



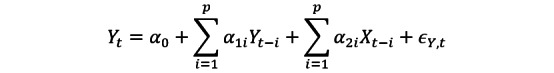



**(3)**



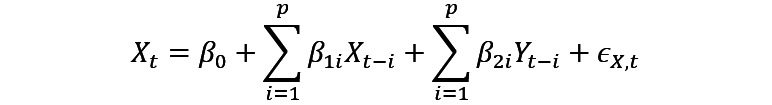



**(4)**

*Y_t_* is the number of suicides at time *t*. *X_t_* is the causal variable (the number of searches for the keyword on Google) at time 𝑡.

Adjustments to the number of suicides in the school calendar model are as follows:

The Poisson regression model takes the following form:


* log(λ_t_)=*γ*_0_+*γ*_1_D_spring,t_+*γ*_2_D_summer,t_+*γ*_3_D_winter,t_*

**(5)**

*λ_t_* is the expected number of suicides at week *t*. *D_spring,t_*, *D_summer,t_*, and *D_winter,t_* are dummy variables at the end of the spring, summer, and winter breaks, respectively. The actual number of suicides data is assumed to follow a Poisson distribution. By subtracting the predictions made by the Poisson regression model from the measured data, we generated suicide count data adjusted for the effects of the school calendar.

After performing the Granger causality test, as other past studies did, we also calculated the cross-correlation coefficients based on the same method as in previous studies [22]. Correlation coefficients with the time lag data were estimated, and a significance test was performed. We assumed that the variance was equivariant when the correlation coefficient was obtained. In this case, the square root transformation was used for the variance stabilization transformation owing to the data frequency. Finally, search terms and suicide frequencies that showed significant differences in the number of suicides were plotted against the number of weeks in 2020 to determine social conditions during the COVID-19 pandemic. All statistical analyses were performed using R (version 4.1.0; The R Foundation), and the significance level was set to 5%.

### Ethical Considerations

Our study was approved by the Research Ethics Review Committee of Japan Suicide Countermeasures Promotion Center on September 16, 2021 (R03-01). Patient consent was waived because this study was a secondary analysis of existing data. The data were made available for research use by processing them on a weekly basis to prevent personal identification.

## Results

### Distribution of the Number of Suicides

The total number of suicides among elementary, middle, and high school students, from January 3, 2016, to December 26, 2020, was 1925 (mean 7.40, SD 3.42). The 52 weeks were divided into 5 periods. The summary statistics for each period were as follows: period 1 (January 3, 2016, to December 31, 2016) was 318 (mean 6.12, SD 2.94), period 2 (January 1, 2017, to December 30, 2017) was 352 (mean 6.77, SD 2.95), period 3 (December 31, 2017, to December 29, 2018) was 366 (mean 7.04, SD 2.81), period 4 (December 30, 2018, to December 28, 2019) was 394 (mean 7.58, SD 3.14), and period 5 (December 29, 2019, to December 26, 2019) was 495 (mean 9.52, SD 4.18). During our study period (260 total weeks), there were 0 weeks with no suicide deaths among elementary, middle, or high school students.

### Evaluation Outcomes

The study variables were compiled and are presented in Table 1. They consist of 11 school-related terms and 9 terms integrated with school words, generating a total of 20 variables.

Unit root tests (ADF) were performed on the original series of suicide variables, all of which exhibited stationary behavior (Table 2). The test results are available upon request from the authors. Using the AIC, a VAR model was selected for Granger causality analysis. The results showed a statistically significant Granger causality between the search terms “I do not want to go to school” (*P*=.006) and “study” (*P*=.02) and the number of suicides (Table 3). The other search terms did not show Granger causality in either direction for the number of suicides.

**Table 1:** The Google Trends search terms selected as the subjects of analysis.

English equivalent search terms			Original Japanese search terms
**Search terms combined with school**			
	school	学校 (gakkou)	
	school reopening	学校 再開 (gakkou saikai)	
	I do not want to go to school	学校 行きたくない (gakkou ikitakunai)	
	school hard	学校 辛い (gakkou tsurai)	
	school anxiety	学校 不安 (gakkou fuan)	
	When is school?	学校 いつ (gakkou itsu)	
	When does school start?	学校 いつから (gakkou itsukara)	
	school start	学校 始まる (gakkou hajimaru)	
	school bullying	学校 いじめ (gakkou ijime)	
**School-related search terms**			
	school friends	学校 友人 (gakkou yuujin)	
	school grades	学校 成績 (gakkou seiseki)	
	bullying	いじめ (ijime)	
	friendships	友人関係 (yuujinkankei)	
	study	勉強 (benkyou)	
	anxiety about studying	勉強 不安 (benkyou fuan)	
	I do not know how to study	勉強 わからない (benkyou wakaranai)	
	mid-term or term-end exam	試験 (shiken)	
	entrance examination	受験 (juken)	
	future career	進路 (shinro)	
	grades	成績 (seiseki)	

**Table 2:** Results of augmented Dickey-Fuller test on time-series data.

Variables	ADF^a^ test statistics	*P* value
the number of suicides	–4.61	<.001
school	–3.91	.01
school reopening	–3.91	.01
I do not want to go to school	–6.72	<.001
school hard	–5.96	<.001
school anxiety	–4.89	<.001
When is school?	–3.49	.04
When does school start?	–4.21	<.001
school start	–4.08	<.001
school bullying	–5.22	<.001
school friends	–5.38	<.001
school grades	–15.67	<.001
bullying	–4.94	<.001
friendship	–6.15	<.001
study	–5.77	<.001
anxiety about studying	–5.45	<.001
I do not know how to study	–6.37	<.001
mid-term or term-end exam	–5.84	<.001
entrance examination	–4.67	<.001
future career	–4.53	<.001
grades	–3.69	.03

^a^ADF: augmented Dickey-Fuller.

**Table 3:** Temporal pre-post relationship by Granger causality (Google Trends model).

Null hypothesis	Optimal VAR^a^ lag length	*F* test (*df*)	*P* value
“school” does not Granger-cause the number of suicides	7	1.10 (7, 476)	.36
The number of suicides does not Granger-cause “school”	7	0.63 (7, 476)	.74
“school reopening” does not Granger-cause the number of suicides	9	0.79 (9, 464)	.63
The number of suicides does not Granger-cause “school reopening”	9	1.32 (9, 464)	.22
“I do not want to go to school” does not Granger-cause the number of suicides	1	7.55 (1, 512)	.006
The number of suicides does not Granger-cause “I do not want to go to school”	1	3.79 (1, 512)	.05
“school hard” does not Granger-cause the number of suicides	1	0.01 (1, 512)	.94
the number of suicides does not Granger-cause “school hard”	1	1.51 (1, 512)	.22
“school anxiety” does not Granger-cause the number of suicides	1	0.94 (1, 512)	.33
the number of suicides does not Granger-cause “school anxiety”	1	0.39 (1, 512)	.53
“When is school?” does not Granger-cause the number of suicides	3	0.19 (3, 500)	.90
the number of suicides does not Granger-cause “When is school?”	3	0.21 (3, 500)	.89
“When does school start?” does not Granger-cause the number of suicides	12	0.93 (12, 446)	.51
the number of suicides does not Granger-cause “When does school start?”	12	0.56 (12, 446)	.88
“school start” does not Granger-cause the number of suicides	3	0.69 (3, 500)	.56
the number of suicides does not Granger-cause “School start”	3	0.74 (3, 500)	.53
“school bullying” does not Granger-cause the number of suicides	1	2.80 (1, 512)	.09
the number of suicides does not Granger-cause “school bullying”	1	0.13 (1, 512)	.72
“school friends” does not Granger-cause the number of suicides	1	0.11 (1, 512)	.74
the number of suicides does not Granger-cause “school friends”	1	1.42 (1, 512)	.23
“school grades” does not Granger-cause the number of suicides	2	0.32 (2, 506)	.73
The number of suicides does not Granger-cause “school grades”	2	1.72 (2, 506)	.18
“bullying” does not Granger-cause the number of suicides	2	1.85 (2, 506)	.16
the number of suicides does not Granger-cause “bullying”	2	0.75 (2, 506)	.47
“friendship” does not Granger-cause the number of suicides	1	0.02 (1, 512)	.89
the number of suicides does not Granger-cause “friendship”	1	0.07 (1, 512)	.79
“study” does not Granger-cause the number of suicides	5	2.63 (5, 488)	.02
the number of suicides does not Granger-cause “study”	5	0.54 (5, 488)	.74
“anxiety about studying” does not Granger-cause the number of suicides	4	0.39 (4, 494)	.82
the number of suicides does not Granger-cause “anxiety about studying”	4	0.40 (4, 494)	.81
“I do not know how to study” does not Granger-cause the number of suicides	1	0.08 (1, 512)	.77
the number of suicides does not Granger-cause “I do not know how to study”	1	0.26 (1, 512)	.61
“mid-term or term-end exam” does not Granger-cause the number of suicides	3	0.27 (3, 500)	.85
the number of suicides does not Granger-cause “mid-term or term-end exam”	3	0.39 (3, 500)	.76
“entrance examination” does not Granger-cause the number of suicides	5	0.86 (5, 488)	.51
the number of suicides does not Granger-cause “entrance examination”	5	0.58 (5, 488)	.71
“future career” does not Granger-cause the number of suicides	4	0.74 (4, 494)	.56
the number of suicides does not Granger-cause “future career”	4	0.89 (4, 494)	.47
“grades” does not Granger-cause the number of suicides	2	2.11 (2, 506)	.12
the number of suicides does not Granger-cause “grades”	2	2.07 (2, 506)	.13

^a^VAR: vector autoregressive.

### Robustness of the Result

For the search words that were found to be significant in the above analysis, we conducted the robustness check by generating a dataset that excludes the effect of the school calendar. The reason for this check is to test whether our search keywords remain statically significant after controlling for the effect of the school calendar. If it remains so, it infers that our search keywords have a powerful predicting power for the number of suicides among elementary, middle, and high school students.

The results of the school calendar model rejected the null hypotheses “I do not want to go to school” does not Granger-cause the number of suicides and “study” does not Granger-cause the number of suicides both as in the Google Trends model (Table 4).

**Table 4:** Temporal pre-post relationship by Granger causality (school calendar model in the search terms “I do not want to go to school” and “study”).

Null hypothesis	Optimal VAR^a^ lag length	*F* statistics (*df*)	*P* value
“I do not want to go to school” does not Granger-cause the number of suicides	1	10.91 (1, 512)	.001
The number of suicides does not Granger-cause “I do not want to go to school”	1	2.74 (1, 512)	.10
“study” does not Granger-cause the number of suicides	2	4.01 (2, 506)	.02
the number of suicides does not Granger-cause “study”	2	1.43 (2, 506)	.24

^a^VAR: vector autoregressive.

### Correlation Analysis

Subsequently, variance stabilization transformations were applied to the phrases shown by Granger causality. A cross-correlation analysis, which revealed that the time-series of suicides lagged by 2 weeks concerning both “I do not want to go to school” and “study,” was performed (Table 5).

In this study, the search queries “I do not want to go to school” and “study” showed Granger causality with suicide incidences. The results of the cross-correlation analysis showed significant positive correlations in the range of –2 to 2 for “I do not want to go to school” (highest value at time lag 0, *r*=0.28) and –1 to 2 for “study” (highest value at time lag –1, *r*=0.18), indicating that the search volume increased as the number of suicides increased.

**Figure 2:**
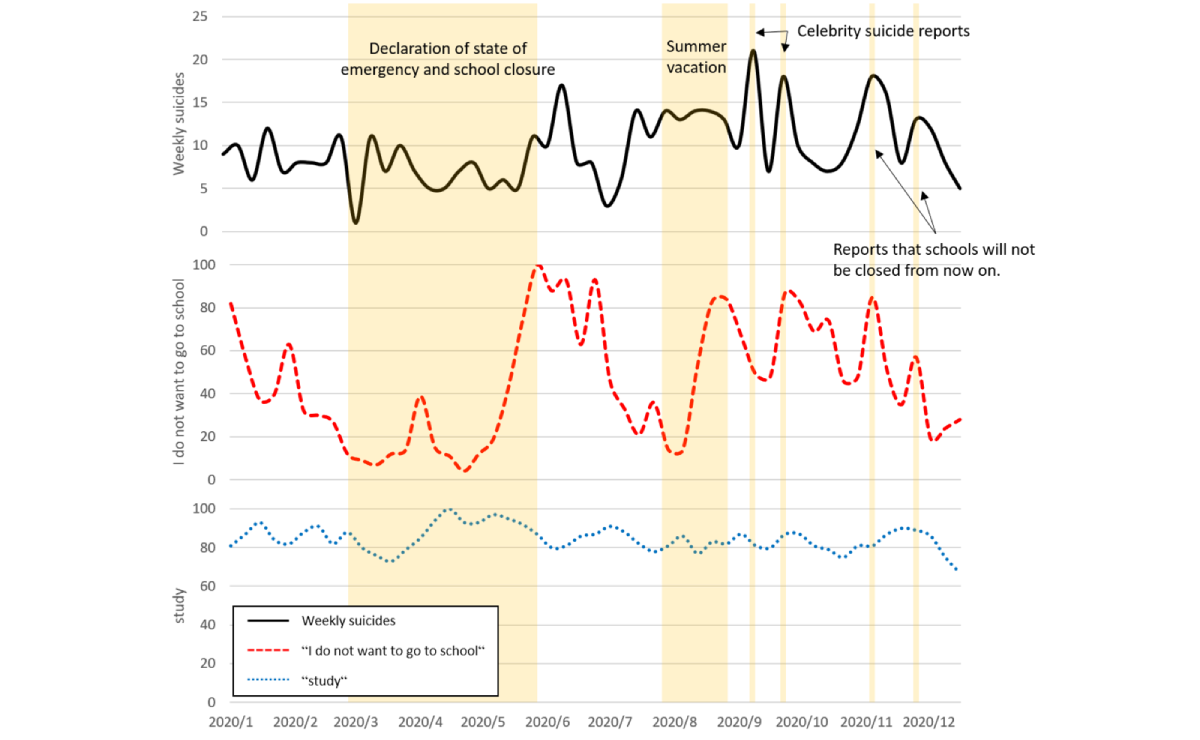
Number of suicides and search queries during the COVID-19 pandemic period. Highlights indicate social conditions. The top line plots weekly suicide, the middle line plots “I do not want to go to school,” and the bottom line plots “study.”.

Furthermore, in 2020, during the COVID-19 pandemic, the time-series trend for “I do not want to go to school” resembled that of the number of suicides. Figure 2 shows the social situation in 2020 and the time-series of the number of suicides and search terms. Both variables decreased from March to May, when schools were closed nationwide, and increased from May to June, when the state of emergency was lifted. This relationship persisted even after the summer vacation and increased from September to October. In addition, 2 more sharp increases were observed after November. Conversely, the search volume for “study” increased from March to May and remained high thereafter, showing no significant change compared with “I do not want to go to school.”

**Table 5:** Cross-correlation with the number of suicides at lags –3 to +3 after variance stabilization.

Search terms	–3	–2	–1	0	1	2	3
“I do not want to go to school”	0.04	0.09^a^	0.21^a^	0.28^a^	0.26^a^	0.17^a^	0.06
“study”	0.05	0.04	0.18^a^	0.17^a^	0.13^a^	0.12^a^	0.09

^a^Statistically significant values at *P*<.05.

## Discussion

### Principal Findings

This study examines the association between the number of suicides among Japanese children and adolescents and the volume of school-related internet searches. We used Granger causality analysis to show that specific school-related internet search volumes may precede the number of suicides among Japanese elementary, middle, and high school students over time. In the Granger causality test, “I do not want to go to school” and “study” were statistically significant in both the Google Trends model and the school calendar model. Internet search terms that were significantly and positively correlated with the number of suicides were “I do not want to go to school” and “study.” Regarding the time lag, a negative direction starting from 0 indicates the periods after the increase in search volumes, whereas a positive direction indicates the periods before the increase in search volumes. Therefore, to investigate whether the number of suicides increases after an increase in search volumes, the negative lag direction should be primarily considered. In this study, lags were calculated weekly, whereas previous studies used monthly aggregates, where a lag of approximately 2-3 months was observed [16,18,22]. However, the lag in this study was less than 2 weeks, indicating the high accuracy of this leading indicator. The results in Table 4 imply that a lag in either direction indicates a short-term association between the search terms and suicide. The positive correlation between the number of suicides and the search volumes suggests that search terms may be a leading indicator of suicide. Furthermore, the volume of searches for “I do not want to go to school” showed a time-series trend similar to the number of suicides among elementary, middle, and high school students, even in the social context of the 2020 COVID-19 pandemic. To the best of our knowledge, our study is the first to investigate the association between the weekly number of elementary, middle, and high school student suicides and the volume of internet searches for school-related terms in Japan. The discovery of new linguistic markers associated with suicide among elementary, middle, and high school students may contribute to research and practice related to suicide prevention. As our analysis is exploratory, aimed at generating hypotheses for future research, we did not apply multiple comparison corrections [29]. Future studies will need to apply more stringent statistical controls when testing these initial hypotheses to validate the findings.

In 2020, during the COVID-19 pandemic, similar time-series trends were revealed between the search volume “I do not want to go to school” and the number of suicides. In Japan, schools were closed nationwide on March 2, 2020, and were reopened when the state of emergency was lifted on May 25, 2020. During this period, a sharp increase was noted in both the number of suicides and search volumes, which could be attributed to the increasing tension over the school’s reopening. This trend could also be observed during the summer vacation from August to September. The increase in the number of suicides in September cannot necessarily be explained solely by the psychological burden of the resumption of school, since reports of celebrity suicides occurred. However, the number of searches for “I do not want to go to school” gradually began to increase before school resumed in September. This phenomenon may be attributed to an increase in the number of searches considered as suicide risk owing to the timing of the end of the long vacation. Therefore, this result may have reinforced the “broken promise effect” theory [30], which suggests that suicide rates tend to increase after a long break from school.

Other negative school-related terms searched during the same period did not yield significant Granger causality or correlation coefficients. This suggested the robustness of the “I do not want to go to school” term being the high-risk keyword for suicide among elementary, middle, and high school students.

The 2 peaks in search volumes in November 2020 might have been influenced by press conferences conducted by the Ministry of Education, Culture, Sports, Science, and Technology on November 10 and November 27, which stated that entrance examinations and the nationwide closure of all schools would not be canceled or requested [31,32]. This statement may have recalled the psychological stress children and adolescents feel about schools. Therefore, government statements related to schools should be made with caution.

This study’s findings are consistent with those of previous studies. A meta-analysis and systematic review indicated that school absenteeism and dropout rates are associated with an increased risk of self-harm and suicide attempts [33,34], suggesting that educational factors may influence suicide. The search term “I do not want to go to school” in this study may be precisely a proxy variable for measuring educational factors such as expectations and the stress of attending school. The results imply that suicide is closely related to the educational system, especially the opening and closing of schools. In other words, it is possible that school opening and closing are positively and negatively correlated with suicide risk, respectively. However, caution must be exercised while interpreting these results. This is because it has been reported that school ties may be a powerful protective factor against youth suicide [35].

They should enable children who do not want to go to school to continue their learning at home or in other settings by providing them with extensive support. For example, the government could provide financial support to encourage participation in alternative education programs. This support could include subsidizing the cost of accessing online learning platforms or providing the necessary funding to attend free schools. Schools should also adopt more flexible scheduling to motivate students to learn. This allows students to progress at their own pace and helps them to balance their academic and personal interests and activities. In addition, mental health support in schools and communities needs to be strengthened to help children for whom school is a distressing experience. The findings of this study contribute to a better understanding of support strategies for children who do not want to go to school and to proposals for promoting comprehensive change in the education system.

In a study in South Korea on suicide-related internet search terms among children and adolescents, the search volume for “dropout” showed a significant positive correlation [36], indicating that school-related search terms were associated with the number of suicides, which is consistent with our findings. However, our study differs in that we used weekly data rather than daily data, which would be affected by each day of the week, Monday through Sunday. Furthermore, our study fills a gap in previous studies because it shows the potential to be used as a risk factor not only in normal times but also in emergencies such as school closures associated with the COVID-19 pandemic.

This study’s strength is exhibited in the process of selecting search terms through an exploratory approach. The internet search terms selected in this study were chosen through an exploratory review of official sources on suicide in Japan. The selection process did not follow a rigorous and systematic methodology. However, relying solely on a systematic list of search terms based on previous research does not necessarily uncover search terms that are effective for suicide prevention. One study hypothesized that internet searches related to past orientation would be positively related to suicide rates because people with regret and depression are more likely to dwell on the past [37]. In another study, from a clinical psychology perspective, search terms associated with absolutist thinking, such as “completely” and “totally,” were used [38]. These emerging linguistic markers may not have been identified through standardized and broad-ranging methodologies. Although we could have selected specific “words” related to suicide, it may have been difficult to select the sentence “I do not want to go to school.”

Online gatekeeping reduces suicidal ideation [39]. However, “Suicide Prevention Results” are not currently implemented for the search term “I do not want to go to school” in the Japanese Google search system. It is believed that whether a helpline is displayed depends on the language and search terms used [40,41]. Our study has the potential to improve the suicide prevention results for children and adolescents and enable effective referrals to counseling services.

Suicide surveillance in Japan has been delayed for more than 6 months [42]. Monitoring high-risk suicide keywords will help improve such delays and aid in rapid administrative support and mental health planning at the national and municipal levels. Furthermore, monitoring and evaluating surveillance systems involves not only promptness but also cost-effectiveness factors [43]. The Google Trends data used in this study can be obtained in real time and free of charge and could complement existing surveillance systems. This study’s results may provide useful information for many stakeholders, such as schools, internet service providers, governments, and municipalities.

### Limitations

This study has several limitations. First, the Google Trends dataset may be susceptible to noise, as search volumes can be influenced by external factors such as social conditions and media coverage, independent of the searcher’s characteristics. In addition, Google Trends has been criticized for its inability to capture user demographics such as age and gender [7]. Our study assumes that the use of school-related search terms may limit the demographics of searchers to younger age groups. Indeed, future experimentation is needed to determine the exact demographic distribution of individuals who perform school-related searches.

Second, an increase in search volumes for specific keywords does not always correspond to an increase in the number of suicides. Some search terms are accompanied by online helpline advertisements in the browser, which may reduce suicide risk by guiding individuals to seek professional help. Consequently, the correlation between internet queries and the number of suicides may be nullified.

Third, the data used in this study only observes search behavior. Recent forms of social media, such as Twitter (rebranded as X) and Instagram (Meta), offer the distinct feature of allowing individuals to express their thoughts and situations, which diverges from searching behavior [24]. Consequently, these new media forms are currently being investigated regarding adolescent suicides [44]. In the future, it will be necessary to not only continually observe search terms through Google Trends but also scrutinize new media that enable the expression of psychological states.

Finally, while this study is concentrated on suicide among elementary, junior high, and high school students in Japan, yielding findings specific to this demographic within the Japanese context, the global availability of Google Trends and suicide data presents an opportunity for broadening the research scope. Therefore, future studies are planned to explore the applicability of these findings across different regions and cultures, aiming to understand the universality and limitations of our conclusions.

### Conclusions

This study identifies an association between the volume of internet searches for school-related terms such as “I do not want to go to school” and “study,” and the number of suicides among children and adolescents in Japan. By analyzing the search volume for school-related terms, which have not received much attention in previous studies, the study suggests the possibility of improving online helpline displays for children and adolescents. The volume of searches for school-related terms may also be useful in monitoring suicide risk among children and adolescents, not only during normal times but also during emergencies such as school closures. However, because internet search volume includes external factors unrelated to suicidal characteristics, it may not be easy to generalize the findings and incorporate them into national and municipal suicide prevention strategies. Further empirical studies will be needed in the future.

## Abbreviations

AIC: Akaike’s information criterion
ADF test: augmented Dickey-Fuller test
RSV: relative search value
VAR: vector autoregressive

## Data Availability

Data supporting this study’s results require permission from the Japan Center for Suicide Prevention. Requests to access these datasets should be directed to TA, the corresponding author.
